# Influence of differing levels of concentrate on circulating cytokine concentrations in beef heifers

**DOI:** 10.1093/tas/txae089

**Published:** 2024-06-02

**Authors:** Erin L Stockland, Molly S Smith, Autumn T Pickett, Reinaldo F Cooke, Rebecca K Poole

**Affiliations:** Department of Animal Science, Texas A&M University, College Station, TX 77843, USA; Department of Animal Science, Texas A&M University, College Station, TX 77843, USA; Department of Animal Science, Texas A&M University, College Station, TX 77843, USA; Department of Animal Science, Texas A&M University, College Station, TX 77843, USA; Department of Animal Science, Texas A&M University, College Station, TX 77843, USA

**Keywords:** cytokine, concentrate, forage, heifers, immune

## Abstract

Components of the immune system (e.g., cytokines and chemokines) can influence reproductive efficiency. Characterizing the influence nutrition has on shifts in circulating cytokine concentrations will allow for a better understanding of reproductive efficiency in beef cattle. This study aimed to determine the effect of diet composition on circulating cytokine concentrations of beef heifers. Using a 3 × 3 Latin square design, pubertal *Bos taurus*-influenced rumen-cannulated heifers (*n* = 15) were fed a diet based on different concentrate percentages. The treatment period consisted of 28-d feeding periods with a washout interval of 21 d. Treatment groups were fed 100% grass hay (high forage; HF), 60% grass hay with 40% corn-based concentrate (intermediate; INT), and 25% grass hay with 75% corn-based concentrate (high grain; HG). Heifers were offered 2% of their body weight in feed daily. Blood was collected on days 0 and 28 of the treatment period for cytokine analysis. Plasma cytokine concentrations were quantified using RayBiotech Quantibody Bovine Cytokine Array Q1 kit according to manufacturer instructions. Concentrations of interferon gamma-induced protein 10 (IP10) linearly decreased with an increased concentrate diet (*P* = 0.037). Concentrations of IP10 differed for heifers consuming HF diet vs. HG diet (3,069.52 vs. 1,001.84 ± 669.01 pg/mL, respectively) and heifers consuming INT diet vs. HG diet (2,886.77 vs. 1,001.84 ± 669.01 pg/mL, respectively); however, there were no significant differences in IP10 concentrations between HF and INT heifers. There was a tendency for interleukin-1 family member 5 (IL1F5) concentrations to be lower for heifers consuming the HG diet compared to INT diet (*P* = 0.08). Results suggest that heifers consuming a high-concentrate diet have lower concentrations of IP10 and IL1F5. Additional research is necessary to better understand the dietary influence on the immune system in developing heifers.

## Teaser

Exploring the influence nutrition poses on the immune system may provide a better understanding of its impact on the reproductive performance of beef cattle. Differing diet composition (high forage vs. high concentrate) may be associated with changes in circulating cytokine concentrations in beef heifers.

## Introduction

With a projected global population of over 9 billion people by 2050, global food demand is expected to rise by 50% to 60% (Falcon et al., 2022). Ensuring that beef producers can keep pace with the rise in demand is essential to the sustainability of the industry. Reproductive efficiency in cow-calf operations ensures that demand is met for the remaining sectors of the beef industry (Diskin and Kenny, 2016). The nutritional status of beef cattle is a known factor that impacts reproductive efficiency (Rae et al., 1993; Hess et al., 2005; D’Occhio et al., 2019). Specifically for developing heifers, higher levels of nutrition and adequate weight gain can result in earlier onset of puberty and achieving target body weight (BW; 65% mature BW) prior to the first breeding season; thus, improving lifetime reproductive efficiency (Fleck et al, 1980; Patterson et al., 1992; Freetly et al., 2011). Additionally, it has been suggested that nutritional status of developing heifers can also impact immune responses (Ault-Seay et al., 2021).

Cytokines and chemokines are components of the immune system and are communication proteins that interact with various cells in an immune response. Cytokines can be classified as either having pro-inflammatory or anti-inflammatory effects (Dinarello, 2000; Zhang and An, 2007). Pro-inflammatory cytokines, such as interleukin (IL)1 and tumor necrosis factor (TNF), promote inflammation and a fever response whereas anti-inflammatory cytokines, such as IL10 and IL13, reduce inflammation and promote healing (Dinarello, 2000). Additionally, cytokines can be pleiotropic, such as IL6, and exhibit characteristics of both pro-inflammatory and anti-inflammatory depending on environmental conditions (Kishimoto, 2006). Chemokines are a smaller class of chemotactic cytokines that can influence the movement of immune cells (Hughes and Nibbs, 2018), and are part of the innate immune system providing a first line of defense against infection (Sokol and Luster, 2015).

The impact of the immune system on reproductive performance has become a growing field of study. Inflammation plays a role in reproductive performance that can impact pregnancy establishment and maintenance (Ribeiro and Carvalho, 2017). In beef heifers classified as either high or low fertility, based on pregnancy establishment post-artificial insemination, inflammation was a major pathway identified among differentially expressed genes in the uterus (Killeen et al., 2014). Moreover, Ault-Seay et al. (2021) noted an increase in intrauterine cytokines following protein supplementation in beef heifers. Potentially, a tailored feeding program could influence and promote certain cytokine populations that would increase reproductive efficiency (Grimble, 1998).

Most cow-calf operations function based on a high to intermediate forage diet with supplemented grain depending on producer goals or forage availability. Understanding immune changes to differing diets ranging from high forage (HF) to high grain (HG) would allow for a greater understanding of the circulating cytokine concentrations and the ability to influence immune response through nutrition. Being able to alter immune responses through diet would provide an avenue for increasing reproductive efficiency in developing heifers. Therefore, the aim of this study was to characterize circulating cytokine profiles of beef heifers consuming different dietary profiles (i.e., forage-based to grain-based diets) to better understand the influence of nutrition on pro-inflammatory and anti-inflammatory cytokine concentrations.

## Materials and Methods

This study was conducted at Texas A&M University O.D. Butler, Jr. Animal Science Complex Nutrition and Physiology Center in College Station, TX, from November 2020 to February 2021. All experimental procedures were approved by the Texas A&M University Institutional Animal Care and Use Committee (IACUC #2018-0099).

### Animals and Treatments

Details of animals and dietary treatments for this experiment were described by Pickett et al. (2022). Briefly, rumen-cannulated, pubertal *Bos taurus*-influenced heifers (*n* = 15) were used for this experiment in which a 3 × 3 Latin square design was implemented with three treatment periods of 28 d and washout intervals of 21d between periods. During each treatment period (days 0 to 28), heifers were housed inside in individual pens (2 × 4 m) with *ad libitum* access to water and fed their respective diet for each period at 2% of their BW. At the beginning of each period (day 0), groups were assigned to receive one of the following treatments: (1) 100% grass hay/(**HF**: *n* = 15), (2) 60% grass hay + 40% corn-based concentrate (30% dried distillers’ grains and 10% cracked corn) or intermediate (**INT**: *n* = 15), or (3) 25% grass hay + 75% corn-based concentrate (60% dried distillers’ grains and 15% cracked corn) of (**HG**: *n* = 15). Heifer diets were designed to be isonitrogenous across treatment groups (14.2% crude protein); however, diets were not isocaloric. Net energy for maintenance (Mcal/kg) was 1.16, 1.52, and 1.85 for HF, INT, and HG, respectively. Net energy for growth (Mcal/kg) was 0.594, 0.902, and 1.21 for HF, INT, and HG, respectively. Rumen pH was measured 4 h post-feeding. Previous research has shown that circulating cytokine concentrations could be associated with changes in hormone profiles during the estrous cycle (Ault-Seay et al., 2021; Smith et al., 2023). Therefore, during the treatment period, heifers received 0.5 mg/d of melengestrol acetate (MGA) to mimic low levels of progestin and suppress ovulation and estrus expression. Throughout the washout interval, heifers were housed in an outside paddock and received ad libitum access to water and grass hay. In the final 3 d of the washout interval, an intramuscular injection of prostaglandin F2α (Lutalyse, 5 mL; 5 mg/mL; Zoetis Animal Health, Troy Hills, NJ) was administered to regress any corpus luteum present before heifers were returned to the individual pens for adaptation.

### Blood Sampling and Cytokine Assay

Blood samples were collected on days 0 and 28 via coccygeal venipuncture into 10 mL sterile lithium heparin vacutainer tubes (BD Vacutainer, Becton, Dickinson, and Company, NJ, USA). Samples were immediately transferred on ice to the lab before being centrifuged at 1,500 *g* for 20 min at 4 °C. Plasma was transferred into 1.5 mL tubes and stored at -20°C until cytokine analysis.

Cytokine analyses were conducted using the Multiplex Quantibody Bovine Cytokine Array Q1 kit (RayBiotech Life, Inc., Peachtree Corners, GA) as previously described by Smith et al. (2023). Concentrations of interleukin (IL)13, IL1α, IL1F5, IL21, interferon (IFN)α, IFNγ, TNFα, chemokine ligand (CXCL)9 or monokine induced by gamma, CXCL10 (interferon gamma-induced protein 10 [IP10]), and macrophage inflammatory protein1b are measured in the Bovine Cytokine Array Q1 kit. Plasma samples were diluted 1:1 as suggested by the manufacturer, and days 0 and 28 samples were assayed. Assays were run per the manufacturer’s instructions, with one standard glass slide being divided into 16 wells (one heifer sample or standard per well) and cytokines are arrayed in quadruplicate within each well. Slides are stored in a dark box at 4 °C before shipping to the manufacturer for laser scanning and data extraction. Absorbance values were recorded as “F532 Medium to B532” by the manufacturer and concentrations were determined using a software (Q-Analyzer Software for QAB-CYT; RayBiotech, Inc., Peachtree Corners, GA, USA). Concentrations are reported in pg per mL for each individual cytokine.

### Statistical Analysis

Data were analyzed as a 3 × 3 Latin square PROC MIXED of SAS 9.4 (SAS Institute, Cary, NC) using heifer as the experimental unit. The model included treatment and period as independent variables, and all results from day 0 were included as an independent covariate for cytokine concentration analysis. All data were analyzed with heifers as a random variable. Orthogonal contrasts were used to partition-specific treatment effects. Contrast statements included were: (1) linear effect of treatment and (2) quadratic effect of treatment. For pairwise comparisons, the ADJUST = TUKEY option was included in the LSMEANS statement. All results are reported as least square means ± standard error. Significance was set at *P *< 0.05, and tendencies were 0.05 ≤ *P *≤ 0.10.

## Results

Cytokine results by dietary treatment are presented in Table 1. Circulating concentrations of IP10 differed between HF and HG groups (3,069.52 vs. 1,001.84 ± 669.01 pg/mL, *P *= 0.04) and between INT and HG groups (2,886.77 vs. 1,001.84 ± 669.01 pg/mL, *P *= 0.05). No differences were reported for IP10 concentrations between HF and INT groups (*P *> 0.05). Additionally, the cytokine IL1F5 tended to have lower concentrations in HG heifers than in INT diet heifers (243.09 vs. 517.07 ± 106.80 pg/mL, *P = *0.08). Concentrations of IFNγ, IL1α, monokine induced by gamma, MIP1b, TNFα, IL13, IL21, and IFNα did not differ by dietary treatment (*P *> 0.05).

**Table 1.** Effects of dietary treatment on the circulating cytokine and chemokine concentrations (pg/mL) in beef heifers

	Treatment^*^				*P*-value	
	HF	INT	HG	SEM	Linear	Quadratic
**IFNα**	253.57	202.15	3.88	143.74	0.153	0.609
**IFNγ**	132.59	156.08	121.15	66.56	0.899	0.704
**IL13**	2,395.12	3,051.36	2,566.28	1,113.69	0.914	0.678
**IL1α**	283.40	355.67	285.18	114.72	0.990	0.550
**IL1F5**	417.20	517.07	243.09	106.80	0.258	0.165
**IL21**	669.20	1,014.26	925.92	445.15	0.685	0.695
**IP10**	3,069.52^a^	2,886.77^a^	1,001.84^b^	669.01	0.037	0.298
**MIG**	1,262.29	1,202.41	1,244.08	283.92	0.953	0.849
**MIP1b**	12.02	29.34	25.50	20.24	0.643	0.671
**TNFα**	3,152.12	2581.17	2,939.27	508.05	0.769	0.460

^*^High forage (HF): 100% grass hay; INT: 60% grass hay + 40% corn-based concentrate; high grain (HG): 25% grass hay + 75% corn-based concentrate.

^a,b^Within row, without a common superscript, indicates significantly different (*P* ≤ 0.05).

## Discussion

Nutritional influence on aspects of immune function has previously been investigated in beef cattle. Ault-Seay et al. (2021) evaluated the influence of protein supplementation on uterine cytokine concentrations in developing beef heifers and observed differences within 28 d of dietary treatment. The present study evaluated changes in circulating cytokine concentrations in heifers receiving increased amounts of a starch-based concentrate after 28 d. Highly fermentable feedstuffs have previously been indicated to upregulate pro-inflammatory cytokine expression in monogastric animals such as swine (Pié et al., 2007). Additionally, HG diets in cattle are associated with low ruminal pH which in extreme cases can result in the release of lipopolysaccharides (LPS) and an inflammatory response (Loor et al., 2016).

Interestingly, the pro-inflammatory chemokine IP10, had lower circulating concentrations in HG heifers. As previously stated, high starch diets have been associated with an increased pro-inflammatory cytokine response. In these heifers, ruminal pH and volatile fatty acid (VFA) profiles were measured and reported by Pickett et al. (2022). As expected, the HG heifers had lower ruminal pH (6.38) compared to HF and INT heifers (6.81 and 6.63, Pickett et al., 2022); however, for ruminal acidosis to be diagnosed, pH is often below 5.5 (Snyder and Credille, 2017). Therefore, this could explain the lack of inflammatory response by HG heifers in the current study. Additionally, IP10 has been growing in use as a marker in human medicine for infectious diseases and general health, functioning as an indicator of infection prior to onset of clinical symptoms (Moreno et al., 2022; Wang et al., 2023). In cattle, IP10 has been suggested as a biomarker for an infection from *Mycobacterium bovis* (bovine tuberculosis; Parsons et al., 2016). Potentially, IP10 levels could provide an avenue for detecting inflammation and immune responses to cattle-fed HG diets, although future research is needed to investigate this possible relationship. Additionally, a limitation of the current study is that diets were not isocaloric. Therefore, it cannot be discounted that changes in cytokine concentrations could be due to differences in caloric intake rather than the amounts of a starch-based concentrate.

One explanation for the decrease in IP10 could be the effect of butyrate on cytokine response. Butyrate, acetate, and propionate are three short-chain fatty acids or VFAs produced through bacterial fermentation during digestion in the forestomach and colon of ruminants. As previously stated by Pickett et al. (2022), these HG heifers exhibited greater butyrate compared to HF and INT heifers. Butyrate is the least abundant VFA, consisting of about 10% of short-chain fatty acids production in cattle (Dijkstra, 1994). However, influences of butyrate on epithelial and immune response have indicated its influences on cytokine concentrations. Butyrate has been observed inhibiting pro-inflammatory cytokine production while promoting anti-inflammatory cytokine production in a human model (Lee et al., 2017). Additionally, butyrate can regulate cell apoptosis, proliferation, and differentiation while also providing anti-inflammatory effects through protection of the digestive barrier (Pryde et al., 2002; Siddiqui and Cresci, 2021). Anti-inflammatory properties are obtained through the inhibition of nuclear factor kappa B (NF-kB), downregulating the gene transcription of pro-inflammatory cytokines (Segain et al., 2000). A diet consisting of a highly digestible concentrate and fiber would allow for an increased production of butyrate leading to a decrease of pro-inflammatory cytokine concentration. Thus, potentially explaining the decrease in IP10 concentrations in the current study. Further investigation into the influence of butyrate on immune function is warranted.

In the current study, there was a tendency for the anti-inflammatory cytokine, IL1F5, concentration to be less in HG heifers compared to INT heifers. Interleukin-1 family members are a group of pro-inflammatory cytokines that serve the innate inflammation by triggering their receptors. IL1F5, otherwise known as IL36 receptor antagonist, blocks pro-inflammatory responses and is primarily located in the skin, and has previously been linked to human skin diseases (Onoufriadis et al., 2011; Dinarello, 2018). IL1F5 concentrations have been shown to fluctuate in response to dietary protein supplementation in beef heifers (Ault-Seay et al., 2021). Moreover, IL1F5 concentrations have previously been negatively correlated with butyrate-producing bacteria including *Butyrivibrio* (Smith et al., 2023) and HG heifers had a greater relative abundance of this bacteria in the rumen (Pickett et al., 2022). Therefore, the increased relative abundance of *Butyrivibrio* in addition to an increase in ruminal butyrate could be associated with the reduced IL1F5 concentrations in HG heifers. However, future studies are necessary to investigate this relationship.

In conclusion, a high-concentrate diet for 28 d appears to impact concentrations of circulating cytokines, specifically IP10 and IL1F5, in developing beef heifers. This is consistent with prior research in which dietary treatments after 28 d can impact cytokine concentrations (Ault-Seay et al., 2021); however, future research could explore cytokine activity within this 28-d period. Moreover, further research is necessary to better understand the dietary influence on the immune system in developing heifers and identify the impacts of subsequent reproductive performance and lifetime reproductive efficiency.
